# Mie resonance-mediated antireflection effects of Si nanocone arrays fabricated on 8-in. wafers using a nanoimprint technique

**DOI:** 10.1186/s11671-015-0865-8

**Published:** 2015-04-03

**Authors:** Eunah Kim, Yunae Cho, Kwang-Tae Park, Jun-Hyuk Choi, Seung-Hyuk Lim, Yong-Hoon Cho, Yoon-Ho Nam, Jung-Ho Lee, Dong-Wook Kim

**Affiliations:** Department of Physics, Ewha Womans University, Seoul, 120-750 South Korea; Department of Nano-Manufacturing Technology, Korea Institute of Machinery and Materials (KIMM), Daejeon, 305-343 South Korea; Department of Physics, KI for the NanoCentury, KAIST, Daejeon, 305-701 South Korea; Department of Chemical Engineering, Hanyang University, Ansan, 426-791 South Korea

**Keywords:** Si, Nanocone array, Antireflection, Mie resonance, Nanoimprint

## Abstract

We fabricated 8-in. Si nanocone (NC) arrays using a nanoimprint technique and investigated their optical characteristics. The NC arrays exhibited remarkable antireflection effects; the optical reflectance was less than 10% in the visible wavelength range. The photoluminescence intensity of the NC arrays was an order of magnitude larger than that of a planar wafer. Optical simulations and analyses suggested that the Mie resonance reduced effective refractive index, and multiple scattering in the NCs enabled the drastic decrease in reflection.

**PACS:** 88.40.H-; 88.40.jp; 81.07.Gf

## Background

Si wafer-based solar cells account for a predominant photovoltaic (PV) market share (90%), although there has been extensive research into devices using various active materials [1]. The advantages of Si as an active material for a PV device include abundance on earth, competitive fabrication cost, and superior device performance. Further efforts to lower the cost to generate electricity have led to light-trapping strategies that improve optical absorption in Si [2-17]. For example, nanostructures exhibit remarkable absorption enhancement, and a graded refractive index and scattering enable desirable antireflection effects [2-13]. The Mie resonance further lowers the optical reflection and increases light absorption [3,4]. The design of proper nanostructures requires understanding of both ray optics and wave optics to realize wide wavelength range and omni-directional absorption enhancement.

Nanoimprint (NI) lithography is one of the strongest candidates for sub-micron lithography techniques as an alternative to conventional optical lithography [18]. State-of-the-art optical patterning has been well established, but the extremely high cost is an obstacle for use in the PV industry. In contrast, the NI technique is beneficial as a low-cost and large-area patterning approach. NI lithography may be limited due to overlay, defects, and template wear because it is based on direct contact and one-to-one pattern duplication; however, solar cell applications have a high tolerance for pattern uniformity and overlay accuracy. Thus, NI lithography is an attractive method for mass production of nanostructured solar cells.

In this article, we report on the fabrication and optical characterization of a Si wafer nanocone (NC) array. NI lithography and subsequent dry etching were used to produce hexagonal NC arrays (height: 470 nm, bottom diameter: 215 nm, center-to-center distance between nearest neighbors: 300 nm) on 8-in. wafers. The NC arrays exhibited dramatically reduced optical reflectance over a broad wavelength range. Photoluminescence (PL) spectra and numerical simulation results showed that Mie resonance in the nanostructures, as well as antireflection effects, significantly contributed to the optical absorption gain.

## Methods

**Figure 1:**
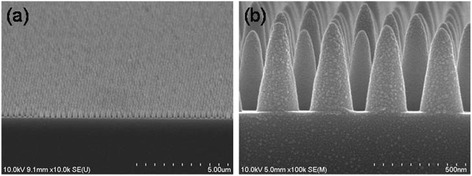
**SEM images of the Si NC array. (a)** Low and **(b)** high magnification.

We fabricated Si NC arrays using NI lithography and subsequent etching on 8-in. Si wafers, as shown in Figure 1. First, the Si master pattern was fabricated by KrF optical lithography (NRS-S203B, Nikon, Shinagawa, Tokyo, Japan), followed by reactive ion etching (Exelan HPT, Lam Research Corporation, Fremont, California, USA), at the National Nanofab Center in Korea. The master pattern had hexagonal arrays of nanopillars, whose diameter and spacing between nearest neighbors were 150 and 300 nm, respectively. The Si master pattern was replicated on a polyurethane acrylate-based custom-made UV-curable resin (PUA) on a polyethylene terephthalate (PET) film, which was used as the NI mold. The PUA consisted of tri-propylene glycol diacrylate (Sigma-Aldrich, St. Louis, MO, USA) and tri-methylol-propane triacrylate (Sigma-Aldrich, St. Louis, MO, USA) as monomers, with 4 wt% 2,2-dimethoxy-2-phenylacetophenone (Sigma-Aldrich, St. Louis, MO, USA) as the photoinitiator and 1 to 4 wt% Rad 2200N (TEGO Chemie Service, Essen, Germany) as the releasing agent. For the UV nanoimprint, a UV-curable resin (mr-UVCur06, Micro Resist Technology, Berlin, Germany) was spin-coated on a Si wafer at 3,000 rpm for 30 s and was subsequently put in contact with the PUA mold under a pneumatic pressure of two bars. Then, the samples were immediately exposed to UV light, and the mold was cautiously released after complete curing of the resin. Residue on the imprinted layer was removed by O_2_ plasma etching before the pattern was transferred to the Si substrate. Finally, the Si NC array was formed by reactive ion etching (TCP-9400, Lam Research Corporation, Fremont, California, USA) under optimized mixing rates of Cl_2_ and HBr at a pressure of 10 mTorr. The morphology of the fabricated NC array was examined using field-emission scanning electron microscopy (SEM; S-4800, Hitachi, Ltd., Chiyoda-ku, Japan). Optical reflection measurements were performed over a wavelength range of 400 to 1,000 nm using an ultraviolet/visible/near-infrared (UV/VIS/NIR) spectrophotometer (Lambda 750, PerkinElmer, Waltham, MA, USA) equipped with a 60-mm integrating sphere (Labsphere Inc., North Sutton, NH, USA) to account for the total light (diffuse and specular) reflected from the samples. For PL measurements, samples were excited by a diode-pumped solid-state (DPSS) laser (wavelength: 532 nm, power: 200 mW), and the luminescence spectra were obtained by an extended InGaAs detector and a lock-in amplifier.

## Results and discussion

**Figure 2:**
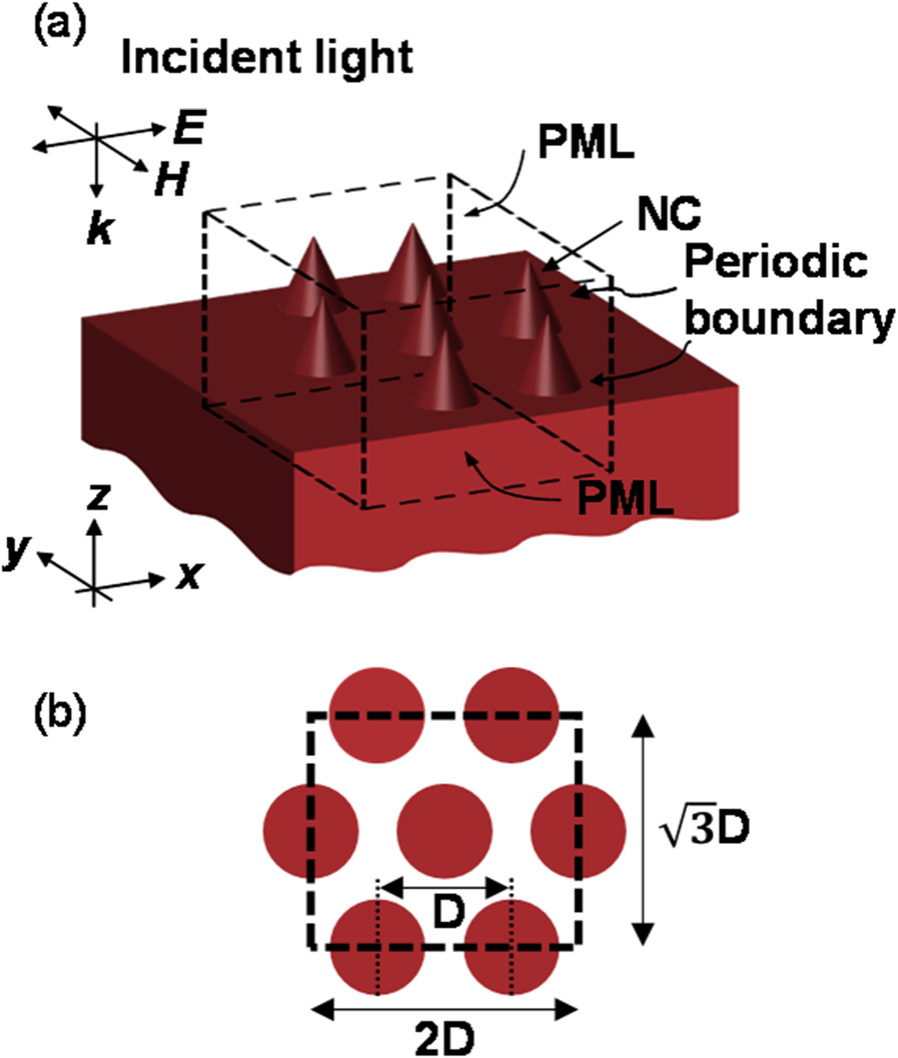
**Schematic diagram of the NC array on an infinitely thick Si substrate. (a)** The volume in the dashed lines indicates the unit cell used in the optical simulation. **(b)** Top view of the unit cell (the spacing between nearest neighbor NCs, *D*, is 300 nm).

Figure 2a shows a schematic diagram of the NC array sample used in our finite-difference time-domain (FDTD) simulations (Lumerical FDTD Solutions). The volume denoted by dashed lines in Figure 2a indicates a repeated unit cell of the hexagonal NC array. In the unit cell, periodic boundary conditions were used at the side walls, and perfectly matched layers (PML) were used as the boundary conditions at the top and bottom surfaces, as illustrated in Figure 2a. The bottom diameter of the NC was 215 nm, the height was 470 nm, and the distance between nearest neighbor NCs (*D*) was 300 nm. Figure 2b shows a top view of the unit cell, as well as the length and width of the rectangular shape. The polarization and propagation directions of the incident light were parallel to the *x*- and *z*-axes, respectively, as illustrated in Figure 2a.

**Figure 3:**
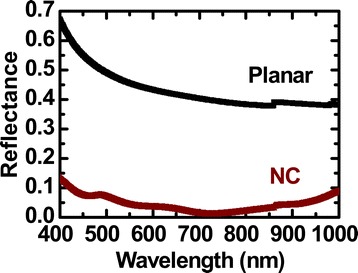
**Experimental optical reflectance spectra.** Comparison of a planar Si wafer and the NC array sample.

Figure 3 shows experimental optical reflectance spectra of a planar Si wafer and the NC array sample. The planar Si exhibited large reflectance (≥38%) over the entire measurement wavelength range due to its large refractive index in the visible range. Such large reflectance and poor absorption due to the indirect bandgap of Si severely limit the energy conversion efficiency of Si solar cells. In contrast, the reflectance of the NC array was less than 10% over a broad wavelength range. Such an antireflection effect is notable, even without a dielectric layer coating [3-5].

**Figure 4:**
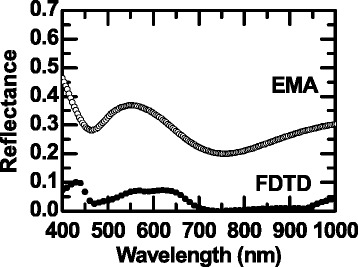
**Calculated optical reflectance spectra of the NC array sample.** Calculated results obtained by the transfer-matrix method using the effective refractive index of NCs based on EMA. FDTD simulation results are shown.

Light will interact with the NC array as a homogeneous medium with a suitable effective refractive index because the dimensions of the NCs are comparable to and less than the wavelength of visible light [11,14]. The effective medium approximation (EMA) theory provides the effective refractive index of the NC array (*n*_eff_): *n*_eff_ = 1.195 to 1.220 for the wavelength range of 400 to 1,000 nm. If the NC array is regarded as a uniform thin layer (thickness = height) with *n*_eff_, the reflectance can be estimated by the transfer-matrix method, as shown in Figure 4 (denoted by EMA) [19]. Figure 4 also shows the reflectance obtained by the FDTD simulation, which is very similar to the experimental data (Figure 3). Broad reflectance dips at approximately 450 and approximately 750 nm were found in both EMA and FDTD reflectance data. The FDTD reflectance, however, was significantly less than the EMA reflectance for the entire calculated wavelength range. At short wavelengths, light will not interact with the NC array as a homogeneous medium. Additional effects (e.g., multiple scattering) can further lower the optical reflectance [11]. The difference between the EMA and FDTD reflectance data was very large, even at long wavelengths where EMA should work. Thus, the notable antireflection effects require physical origins in addition to the aforementioned ones, such as a reduced effective refractive index and multiple scattering.

Spinelli et al. [3] and Wang et al. [4] reported that dielectric nanostructures drastically decrease reflection from a Si wafer with the aid of geometrical Mie resonance. They fabricated Si nanostructure arrays on wafers, whose diameter, height, and period were comparable to those of the NC array used in this study. Hence, similar Mie resonance should play an important role in the significant antireflection effects of our NC array. The experimental reflectance was not identical to the FDTD reflectance over the entire wavelength range, similar to Spinelli et al.’s previous work [3]. The difference could be due to imperfections in the nanopatterns used in the experiments. For example, the height and diameter of the NCs could be slightly different, as shown in Figure 1b, which results in less notable features of Mie resonance. However, the overall reflectance of the samples was comparable to the calculated data, which assumed an ideal regular NC array. Such tolerance of the periodic nanostructures is beneficial for cost-efficient fabrication of nanopatterned solar cells.

**Figure 5:**
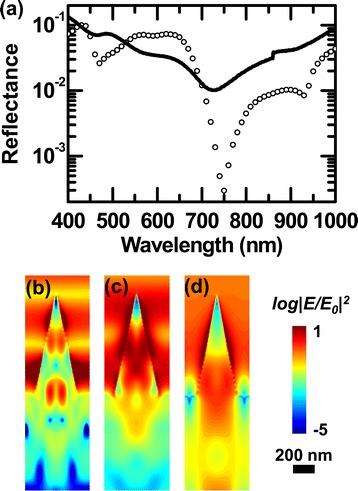
**Reflectance spectra and spatial distribution of the electric field intensity, |** ***E*** **/** ***E***_0_ **|** ^2^ **, of the NC array. (a)** Experimental data (solid line) and FDTD simulation (open symbols) of the optical reflectance of the NC array and a cross-sectional view of the field intensity near the surface of the NC at wavelengths of **(b)** 450 nm, **(c)** 550 nm, and **(d)** 750 nm. *E*_0_ indicates the electric field of the incident light.

Figure 5a shows the optical reflectance spectra of the NC array using a log scale. Local minima of the reflectance appeared at approximately 450, approximately 600, approximately 750, and approximately 950 nm in both the experimental and FDTD simulation results. Such broad dips can be features of Mie resonance [3,4]. A closely spaced NC array has broader resonance behavior because well-separated resonance wavelengths in an isolated NC can merge [4]. Resonance over a wide wavelength range helps increase the energy conversion efficiency of solar cells.

Figure 5b,c,d shows the spatial distributions of the electric field intensity for the NC array sample when illuminated by light with a wavelength of 450, 550, and 750 nm, respectively. The intensity of incident light shows exponential decay in planar Si as the distance from the surface increases; the characteristic length is determined by the optical penetration depth of the light (e.g., 1.56 μm at a wavelength of 550 nm). In contrast, the intensity distribution in the NC array was completely different from that of the planar counterpart; strongly concentrated light intensity appeared in the NCs (Figure 5b,c,d). This clearly shows that Mie resonance dominated the reflection spectra (Figure 5a) and enabled the notable antireflection effects in our NC array samples. As a result, a large portion of the incoming photons was absorbed in the NCs, rather than in the underlying planar part. Surface-concentrated light generates photoexcited carriers near the space charge region, where the built-in potential readily separates the electron-hole pairs. This can boost the carrier collection efficiency of such nanostructure-based solar cells [16,17].

**Figure 6:**
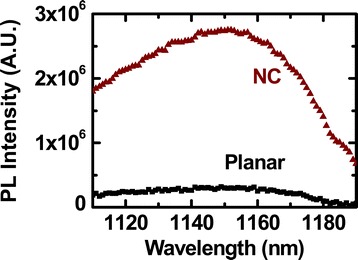
**PL spectra.** A planar Si wafer compared to the NC array sample.

Figure 6 shows the PL spectra of a planar Si wafer and the NC array. The peaks of both spectra appeared at 1,154.8 nm (i.e., photon energy of 1.08 eV), corresponding to the bandgap energy of Si. The PL intensity of the NC array was an order of magnitude larger than that of the planar wafer; the intensity increase was much larger than the surface area ratio between the NC array and the planar sample (2.6). Thus, the enlarged surface area of the NC array alone may not explain the enhanced PL intensity. During the PL measurements, the incoming photons (energy: 2.33 eV) generate electron-hole pairs in Si. The excited electrons will undergo either radiative recombination, generating light emission, or relaxation to surface defect states. We carried out the dry etching process of the planar Si wafer in the same manner as the NC array, and hence, both samples had similar defect state densities at the surface. Thus, the surface area mainly determined the contribution of the defect-mediated recombination to the PL spectra. As shown in Figure 5c, the strongly concentrated light near the surface of the NC array was much larger than that of the planar counterpart. A greater number of photons (light intensity ∝ number of photons) will produce more photoexcited carriers, which could increase the emission light intensity.

## Conclusions

We fabricated antireflection NC arrays on 8-in. Si wafers using the NI technique. NI lithography is an affordable alternative to state-of-the-art photolithography and can be used for mass production during nanofabrication. The optical reflectance of the NC array was less than 10% in the visible range. EMA alone could not explain such a broad range of antireflection effects. The Mie resonance-mediated strong light confinement in the NCs could explain the reduced reflectance and significant enhancement of the PL intensity of the NC array, as supported by optical simulation results.

## Abbreviations

EMA: effective medium approximation
FDTD: finite-difference time-domain
NC: nanocone
NI: nanoimprint
PET: polyethylene terephthalate
PL: photoluminescence
PML: perfectly matched layer
PUA: polyurethane acrylate-based custom-made UV-curable resin
PV: photovoltaic
SEM: scanning electron microscopy
